# Effect of empagliflozin on cytoskeletal repair in the hippocampus of obese mice

**DOI:** 10.3389/fnins.2022.1000839

**Published:** 2022-11-02

**Authors:** Xiaoyi Chen, Liang Ma, Jingyu Zhao, Xiaoyu Pan, Shuchun Chen

**Affiliations:** ^1^Graduate School of Hebei North University, Zhangjiakou, China; ^2^Department of Endocrinology, Hebei General Hospital, Shijiazhuang, China; ^3^Department of Neurology, Hebei General Hospital, Shijiazhuang, China; ^4^Graduate School of North China University of Science and Technology, Tangshan, China

**Keywords:** empagliflozin, hippocampus, high-fat diet, cognitive function, phosphoproteomics analysis

## Abstract

**Objective:**

We aimed to investigate the effect of empagliflozin on hippocampal phosphorylated protein levels in obese mice.

**Materials and methods:**

Sixteen obese mice successfully modeled on high-fat diet were randomly divided into high-fat feeding group (group H) and empagliflozin group (group H + empagliflozin, group E), eight mice in each group, and eight C57BL/6J male normal mice were selected as the control group (normal control, group C). Group E was treated with empagliflozin 10 mg/kg/d for 12 weeks, while mice in groups H and C were treated with equal amounts of saline. The spatial learning memory ability of the mice was determined by the Morris water maze experiment. Further, their body weights and serological indices were measured. Finally, total proteins were extracted from hippocampal tissues for functional analysis by the phosphorylated proteomics method.

**Results:**

The results showed that escape latency was prolonged, retention time in the target quadrant was shortened, and the number of loop penetrations was reduced in the obese mice induced by a high-calorie diet compared with normal controls, whereas escape latency was shortened, retention time in the target quadrant was increased, and the number of loop penetrations was increased after empagliflozin treatment. Phosphoproteomics in the high-fat/control (H/C), empagliflozin/high-fat (E/H), and E/C groups showed 844, 1,552, and 1,512 differentially significant phosphorylation sites, respectively. The proteins corresponding to these differentially phosphorylated sites were mainly involved in neurodegenerative pathways and actin cytoskeleton regulation. Notably, myosin heavy chain 10 (MYH10), p21 protein-activated kinase 4 (PAK4), phosphatidylinositol 3 -phosphate 5-kinase (PIKfyve), and other differentially phosphorylated proteins were involved in actin cytoskeleton regulation.

**Conclusion:**

We concluded that empagliflozin protects cognitive functions by inducing serine phosphorylation in MYH10, PAK4, and PIKfyve in the hippocampal tissue of obese mice.

## Introduction

In recent decades, the number of people with obesity has been growing worldwide at an alarming rate. The latest data released by the World Health Organization shows that nearly 2 billion adults worldwide are overweight or obese, and this figure is still growing (Ponasenko et al., 2022). According to “Dietary Guidelines for Chinese Residents” released in 2022, more than 50% of adults in China are overweight, or obese, making it the country with the largest number of overweight people or people with obesity in the world. Obesity causes various pathophysiological changes, leading to comorbidities such as type 2 diabetes, musculoskeletal disorders, cardiovascular and cerebrovascular diseases, and cancer, of which cognitive dysfunction is prominent comorbidity (Miller and Spencer, 2014). Clinical and experimental evidence suggests that obesity and high-fat diets (HFDs) are associated with deficits in learning, memory, and executive function and potentially brain atrophy (Solas et al., 2017). Epidemiological evidence also suggests that individuals with obesity possess a double risk of Alzheimer’s disease compared with those with normal weights (Yu et al., 2020). Obesity affects cognitive function; however, its associated pathogenesis is sophisticated and not fully elucidated. Known mechanisms include inflammation, blood–brain barrier damage, insulin resistance, and gut flora dysbiosis, all of which affect synaptic and neuronal functions of the brain, ultimately changing cognitive functions in patients with obesity.

Protein post-translational modifications (PTMs) can alter protein structure and activity, mediate cellular signaling, and respond to external environmental stimuli at the cellular level (Lin and Caroll, 2018). Phosphorylation is the most widely studied protein PTM, which regulates biological growth and development, signal transduction, and disease development (Kamacioglu et al., 2021). Evidence suggests that protein phosphorylation is critical for regulating hippocampal signaling pathways such as nitric oxide synthase (Xiao et al., 2019), cell cycle protein-dependent kinase 5 (Im et al., 2022), and mitogen-activated protein kinase (Lee and Kim, 2017) related signaling pathways involved in axon growth, neuronal migration, and synapse formation. Notably, actin cytoskeleton regulation is crucial for preserving the structural and functional integrity of the hippocampus. Mutations or deletions in genes encoding cytoskeleton-associated proteins, comprising myosin heavy chain 10 (MYH10) (Tuzovic et al., 2013) and p21 protein-activated kinase 4 (PAK4) (Putz et al., 2021), can lead to a central nervous system (CNS) phenotype with impaired cognitive functions. In contrast, the loss of phosphatidylinositol 3-phosphate 5-kinase (PIKfyve) function leads to neurodegeneration in mouse models and human patients (Rivero-Rios and Weisman, 2022). However, fewer studies are available on cytoskeletal proteins associated with obesity-induced cognitive impairment.

Sodium–glucose cotransporter 2 inhibitors (SGLT2is) were originally developed for the treatment of type 2 diabetes mellitus (T2DM), and their glucose-lowering mechanism does not depend on improving insulin secretion or resistance, rather they block the improving insulin secretion or resistance as well as exert their glucose-lowering effects by blocking glucose reabsorption from the proximal renal tubule, thus increasing urinary glucose excretion. Large double-blind clinical trials showed that in addition to glycemic control, empagliflozin might also increase life expectancy by reducing cardiovascular mortality (Zinman et al., 2015). In addition, growing evidence indicates the presence of SGLT in the mammalian CNS (Yu et al., 2013). SGLT2 receptors are present in regions such as the hippocampus, cerebellum, and blood–brain barrier endothelium, and this specific distribution may account for the neuroprotective properties of SGLT2 inhibitors (Sa-Nguanmoo et al., 2017). Empagliflozin improved learning and memory in a mixed mouse model of Alzheimer’s disease and type 2 diabetes (Lin et al., 2014). Empagliflozin also improved cognitive function by reducing oxidative stress, astrocyte activation, and inflammation. A case-control study on 11,619 patients with T2DM found that an SGLT2i was significantly associated with a reduced risk of dementia in patients with diabetes (Wium-Andersen et al., 2019). However, to the best of our knowledge, the effect of empagliflozin on cognitive functions in obese mice has not been previously studied using a phosphorylated proteomic approach.

Therefore, we are the first to study the levels of hippocampus-associated phosphorylated proteins in obese mice using a phosphoproteomics approach, to investigate the effect of empagliflozin on the levels of these phosphorylated proteins, and to probe the effect of empagliflozin on cognitive functions in obese mice and its possible mechanisms.

## Materials and methods

### Ethics statement

All experimental procedures were conducted in accordance with the “3R” principle and conformed to the regulations of the Animal Ethics Committee of Hebei General Hospital (Number: 202173).

### Animals

Six-week-old male C57BL/6JC mice (*n* = 24) were purchased from Hebei Ivivo Biotechnology Co., Ltd. (license number: SYXK[Jun] 2015-0004, Hebei, China) and housed in the Animal Experiment Center of Hebei General Hospital at room temperature, humidity, diet, and drinking water availability in accordance with the standard experimental animal feeding conditions. After 7 days of adaptive feeding, eight mice were randomly assigned to the normal group based on their body mass and fed with normal chow (whole family nutritional pellet growth diet; Beijing Huafukang Biotechnology Co., Ltd., Beijing, China) from the beginning to the end of the experiment; the remaining mice were fed with high-fat chow (containing 60% fat; Beijing Huafukang Biotechnology Co., Ltd., Beijing, China) to establish the obesity model. After 12 weeks of modeling, 20% of the average body mass of normal mice was required to have been exceeded to be considered successful in inducing obesity. The qualified obesity model mice were randomly divided into high-fat diet group (group H) and high-fat diet + empagliflozin group (group E) according to body mass, and mice in group E received 10 mg/kg/day of empagliflozin *via* gavage, and the groups C and H mice were provided with an equal amount of saline *via* gavage. The empagliflozin intervention was continued for 12 weeks.

### Mouse behavioral assay

To assess the spatial learning and memory abilities of the mice, a water maze test was performed. For the water maze experiment, a pool was divided into four quadrants and a platform of 10-cm diameter was placed in the third quadrant and placed underwater for 1 cm. The mice were first trained for four consecutive days. The latency period was calculated as the time for the mice to find the platform. If the mice could not find the platform in 60 s, they were guided to find the platform and placed on it for 10 s. The latency period of the training phase was recorded for the control and experimental mice. On day 5, the experimental platform was removed, the mice were placed in water, and the number of times they passed through the platform within 60 s and the proportion of time in the original platform quadrant to the total time was recorded.

### Tissue sampling and processing

After 24 h of the behavioral experiments, the mice in each group were weighed, and the blood was removed from their eyes. The plasma specimens were centrifuged at 3,000 × *g* for 10 min at 4°C, the upper serum was separated, and the serum was aspirated and divided into labeled lyophilized tubes and set aside at −80°C. After the blood sampling, the brain was quickly severed on the freezing table, separated from the bilateral hippocampal region, snap frozen in liquid nitrogen, and stored at −80°C.

### Testing of serum lipids and oxidative stress markers

The serum TG, TC, LDL-C, and HDL-C levels were measured by using a fully automated enzyme labeling assay VERSAmax (USA). The serum SOD activity was measured by xanthine oxidase method and serum MDA content was measured by thiobarbituric acid method.

### Protein purification and sample preparation

The frozen hippocampal tissues were grounded with liquid nitrogen. Protease inhibitor and phosphatase inhibitor were added to each group of the samples, mixed to a final concentration of 1 mm, sonicated on an ice bath for 3 min, centrifuged at 12,000 × *g* for 10 min, and the supernatant was removed. The protein solution was adjusted to 5 mm concentration by adding dithiothreitol and incubated for 30 min at 56°C. Then, iodoacetamide was added to the solution to adjust the concentration to 10 mm and incubated for 15 min at 4°C away from light. Finally, six times the volume of acetone was added to precipitate the proteins, and −20°C was used to precipitate overnight. The precipitate was collected *via* centrifugation at 4°C, 8,000 × *g* for 10 min, and the precipitate was re-solubilized with ammonium bicarbonate (50 mm), added to trypsin at the mass ratio of 1:50 (trypsin: protein), and then digested overnight at 37°C. The peptides were vacuum freeze-dried and used for subsequent analyses.

### Phosphorylated peptide enrichment

Phosphopeptide enrichment was performed using the IMAC phosphopeptide Enrichment Kit (Thermo Fisher Scientific Inc., USA) according to the manufacturer’s instructions (Thermo Scientific). After lyophilization, they were stored at −20°C for subsequent mass spectrometry analysis.

### LC-MS/MS analysis

The phosphopeptides were resuspended in mobile phase A (0.1% formic acid aqueous solution) and separated using the EASY-nLC 1200 UPLC system at a flow rate of 300 nl/min. The mobile phase B was composed of an 80% acetonitrile aqueous solution containing 0.1% formic acid. The peptides were separated by a linear gradient from the abovementioned solution and then injected into the NSI ion source for ionization, followed by placing into the timsTOF Pro mass spectrometer for analyses. The tests were repeated thrice.

### Phosphorylated protein screening and identification

The MS raw data for each sample were combined, and the secondary mass spectrometry data were retrieved using the MaxQuant software. Next, the inverse library was added to calculate the false positive rate of random matches occurring and controlled at <1%. The search parameters were fixed modifications of “Carbamidomethyl (C)” and variable modifications of “Oxidation (M), Acetyl (M), and Protein N-terminal (M).” The tolerance of the first search peptide and MS/MS match were set to 20 ppm and 0.5 Da, respectively, and the maximum missed cleavage was set to 2.

### Bioinformatics analysis

Protein function annotation was performed using different functional databases, principal component analysis (PCA), 10 volcano plots of differentially expressed proteins, and 11 enrichment analyses of Gene Ontology (GO) and Kyoto Encyclopedia of Genes and Genomes (KEGG) pathway were performed as reported previously. Fisher’s exact test for the evaluation of differentially phosphorylated Fisher’s exact test for the evaluation of differentially phosphorylated proteins and the Benjamini–Hochberg correction for multiple testing was further applied to adjust the derived *p*-values. The pathways with *p* < 0.05 were considered to indicate statistical significance.

### Statistical analysis

SPSS 25.0 software was used for statistical analysis, and experimental results were expressed as mean ± SD. Data were analyzed by one-way ANOVA followed by *post-hoc* test using LSD; data that did not satisfy normal distribution were tested by non-parametric test and differences between groups were compared by Bonferroni; For the escape latency of the water maze a repeated measures ANOVA was used. *P* < 0.05 was considered to indicate a statistically significant difference.

## Results

### Empagliflozin reduces body weight and lowers fasting blood glucose levels in obese mice

**FIGURE 1:**
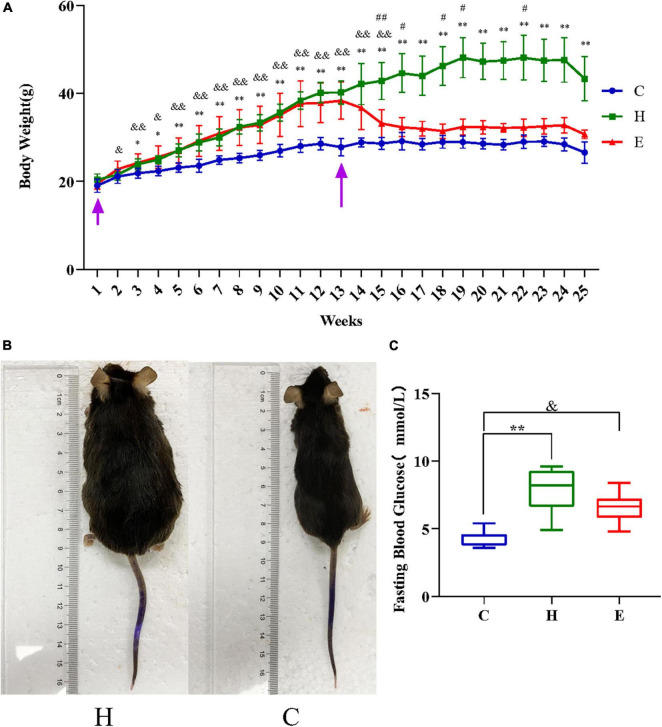
The first arrow on the left in panel **(A)** indicates the time of starting to raise the mice and the second arrow indicates the time of starting the drug intervention. **(A)** Changes in the body weight of mice in the three groups. **(B)** Gross morphology of the mice after successful modeling. **(C)** Comparison of the mean fasting glucose levels in the three groups. *P < 0.05 and ^**^P < 0.01 H vs. C, ^#^*P* < 0.05 and ^##^*P* < 0.01 E vs. H, ^&^*P* < 0.05 and ^&&^*P* < 0.01 E vs. C.

Figure 1A presents the comparison between the changes in the body weights of the mice in the normal diet group (normal diet, C), the HFD group (H), and the empagliflozin group (empagliflozin + HFD, E). As shown in Figure 1A, the initial body weights of the mice in each group were not statistically significant (*P* > 0.05), and the body weights increased incrementally as the rearing time was extended. After 3 weeks, the body weights of the mice in the H group were significantly higher than those in the C group (*P* < 0.05). After 12 weeks, the body weights of the mice in the H group were still significantly higher than those in the C group (*P* < 0.01) and were >20%, reaching the obesity criteria (Figure 1). The body weights of the mice decreased significantly after empagliflozin intervention (*P* < 0.05). Figure 1C presents the comparison between the mean fasting blood glucose levels of the three groups. The results showed that the fasting blood glucose levels of the mice in the H group were significantly higher than those in the C group (*P* < 0.01), whereas the levels of the mice in the E group decreased compared with those in the H group.

### Empagliflozin improves blood lipid levels in obese mice

**FIGURE 2:**
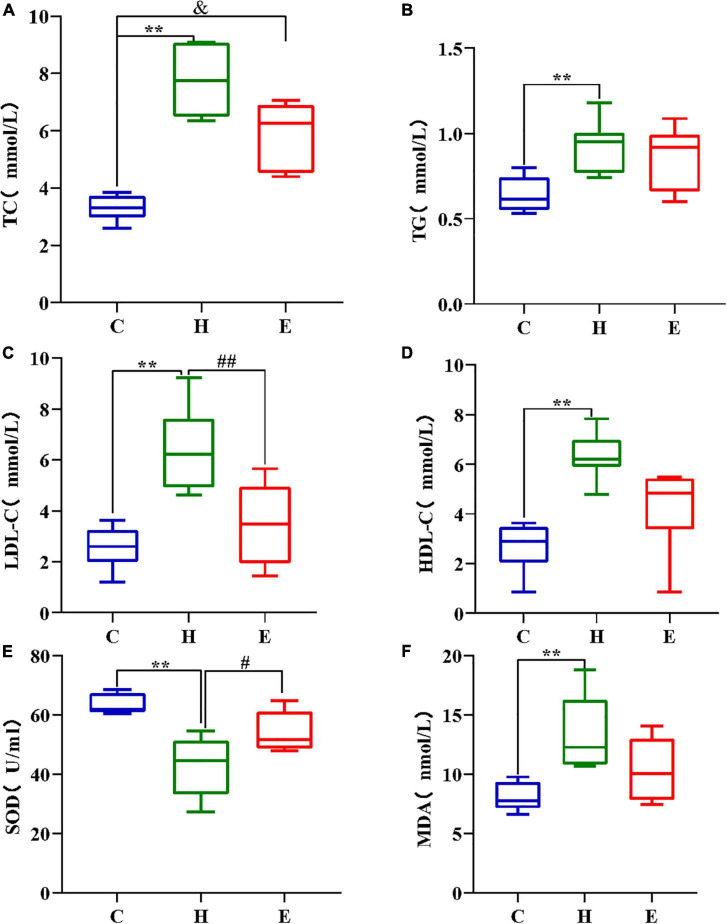
Comparison of TC **(A)**, TG **(B)**, LDL **(C)**, and HDL **(D)** in different groups; **(E,F)** High-fat diet decreased SOD concentration and increased MDA concentration in the H group mice, while the addition of empagliflozin increased SOD concentration and decreased MDA concentration in the group E mice. *P < 0.05 and **P < 0.01 H vs. C, ^#^*P* < 0.05 and ^##^*P* < 0.01 E vs. H, ^&^*P* < 0.05 and ^&&^*P* < 0.01 E vs. C.

As shown in Figures 2A–D, the levels of total cholesterol, triglycerides, low-density lipoprotein, and high-density lipoprotein cholesterol were significantly higher in the H group than those in the C group (all *P* < 0.01); however, these levels were remarkably lower in the E group after empagliflozin intervention than those in the H group.

### Empagliflozin improves oxidative stress levels in obese mice

The levels of SOD, an anti-oxidative stress component, and MDA, a pro-oxidative stress component, were measured to evaluate the differences in serum oxidative stress levels among the three groups. As shown in Figures 2E,F, SOD levels were significantly lower (*P* < 0.01), whereas MDA levels were significantly higher (*P* < 0.01) in the H group than those in the C group. Compared with the H group, the serum levels of SOD and MDA were remarkably increased and decreased, respectively, in the E group. These findings show that a long-term and regular HFD causes excessive oxidative stress in mice, and the administration of exogenous empagliflozin can inhibit and reduce oxidative stress levels.

### Empagliflozin improves cognitive decline in obese mice

**FIGURE 3:**
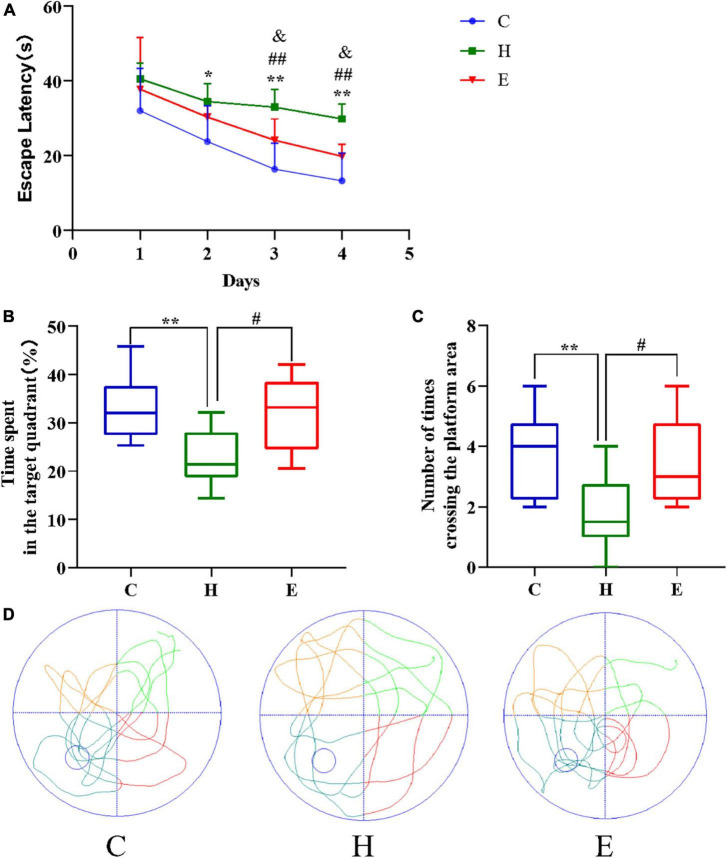
Empagliflozin ameliorates spatial learning and memory deficits induced by a high-fat diet (HFD) in an MWM task. **(A)** Escape latency of different groups of mice during the training phase. **(B)** The duration of mice’s stay in the target quadrant. **(C)** The number of times mice traversed the platform in 1 min. **(D)** Movement trajectories of different groups of mice in the spatial exploration experiment. *N* = 8 per group, *P < 0.05 and **P < 0.01 H vs. C, ^#^*P* < 0.05 and ^##^*P* < 0.01 E vs. H, ^&^*P* < 0.05 and ^&&^*P* < 0.01 E vs. C.

All mice were subjected to the Morris water maze test to assess the status of cognitive functions such as learning and memory. As shown in Figure 3, the escape time of the mice in the H group was significantly longer compared with that in the C group (day 2: *P* < 0.05; day 3: *P* < 0.01; day 4: *P* < 0.01), and the escape time of the mice in the E group was significantly lower compared with that in the H group (day 3: *P* < 0.01; day 4: *P* < 0.01) after an empagliflozin intervention. Similarly, contrasted with the mice in the C group, two indicators, namely, “percentage of time spent in the target quadrant of the original platform” and “the number of times crossing the area of the original platform” were significantly decreased in the H group (*P* < 0.01 for both), whereas in the E group, these two indicators were significantly increased (*P* < 0.05 for both) compared with the H group. These results suggest that the long-term and regular HFD caused relatively considerable cognitive dysfunction in the mice, and the administration of exogenous empagliflozin could improve this cognitive dysfunction.

### Identification of phosphorylated proteins and sites

**FIGURE 4:**
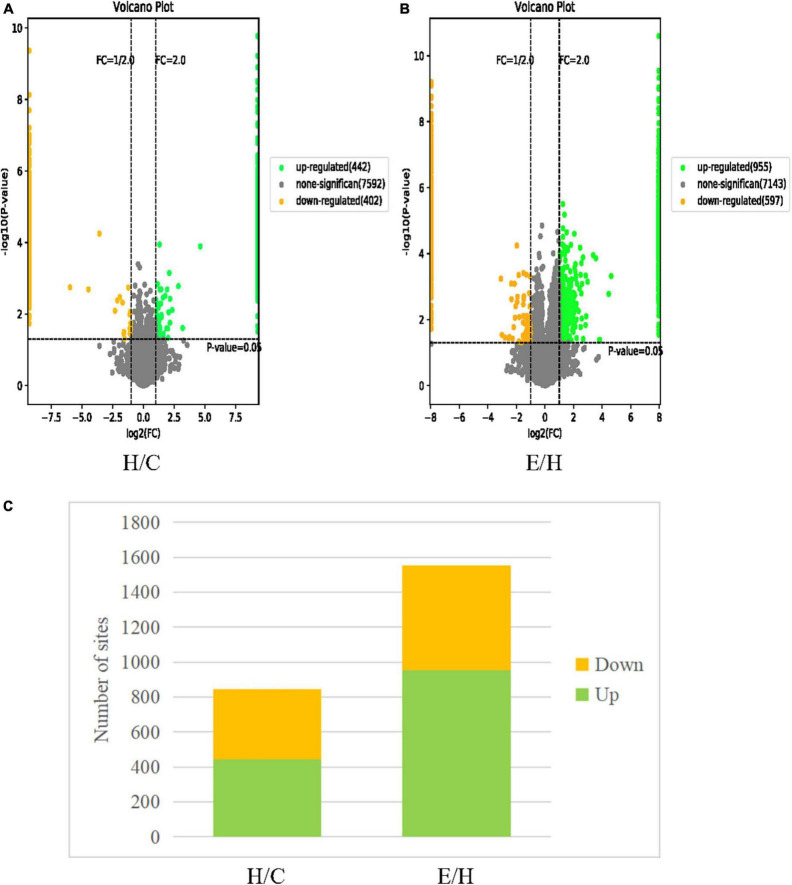
Volcano plots of differentially phosphorylated proteins in **(A)** H/C and **(B)** E/H. The horizontal coordinate represents the relative quantitative value of the protein after log_2_ conversion and the vertical coordinate presents the *P*-value after –log_10_ conversion. Green dots, yellow dots, and gray dots indicate remarkably upregulated, remarkably downregulated, and non-significant differentially expressed proteins, respectively. **(C)** Histograms of the distribution of differentially phosphorylated sites in the H/C and E/H groups. Green and yellow indicate remarkably up- and downregulated phosphorylation sites, respectively.

A total of 7018 phosphorylation sites located on 1,993 proteins were identified by phosphoproteomics analysis. To ensure the credibility of results, the identification data were filtered using the criteria of localization probability >0.75 and delta score ≥8 to obtain phosphorylation sites with high confidence. Then, 2,396 remarkably altered phosphorylation sites were obtained using | fold change| > 2 and *P* < 0.05 as the filtering criteria. Among these altered phosphorylation sites, 844 differentially significant phosphorylation sites in the H/C group, including 442 upregulated and 402 downregulated phosphorylation sites. A total of 1,552 differentially significant phosphorylation sites in the E/H group were obtained, containing 955 upregulated and 597 downregulated phosphorylation sites. These differentially expressed proteins are shown in the volcano plot in Figure 4.

### Enrichment of differentially phosphorylated protein functions

**FIGURE 5:**
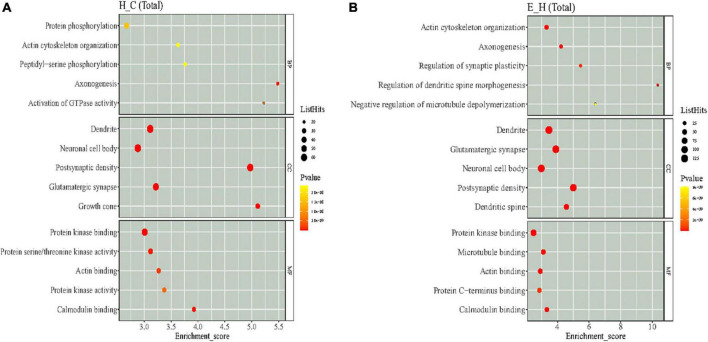
The results of Gene Ontology (GO) enrichment analysis of differentially phosphorylated proteins in the **(A)** H/C and **(B)** E/H groups. The *X*-axis represents the enrichment score, and the *Y*-axis represents the top five term information of each BP/CC/MF. The larger the bubble, the more entries contain the number of proteins corresponding to the differential sites, the color of the bubble changes from yellow to red, and the smaller its enrichment *p*-value value is, the greater its significance.

The functional enrichment of the differentially phosphorylated proteins was mainly performed by GO classification, KEGG pathway to detect whether differential modifications have a remarkable enrichment trend in certain functional types. The GO functions were analyzed at three levels: molecular function (MF), biological processes (BP), and cellular composition (CC), and bubble plots were obtained according to the top 5 rankings of −log_10_ *P*-value corresponding to each level as shown in Figure 5. The GO analysis of the H/C group showed that regarding MF, the differentially phosphorylated proteins were mainly associated with protein kinase binding, protein serine/threonine kinase activity, actin binding, protein kinase activity, and calmodulin binding. Considering BPs, the differentially phosphorylated proteins were involved in protein phosphorylation, actin cytoskeleton organization, peptidyl-serine phosphorylation, axonogenesis, and activation of GTPase activity. In terms of CC, these differentially phosphorylated proteins were distributed in the dendrite, neuronal cell body, post-synaptic density, glutamatergic synapse, and growth cone. The GO analysis of the E/H group showed that regarding MF, the differentially phosphorylated proteins were associated with protein kinase binding, microtubule binding, actin binding, protein C-terminus binding, and calmodulin binding. In terms of BP, the differentially phosphorylated proteins were involved in actin cytoskeleton organization, axonogenesis, synaptic plasticity regulation, regulation of dendritic spine morphogenesis, and negative regulation of microtubule depolymerization. Regarding CC, these differentially phosphorylated proteins were distributed in the dendrite, glutamatergic synapse, neuronal cell body, post-synaptic density, and dendritic spine. The enrichment analysis showed that these differentially phosphorylated proteins were mainly involved in actin cytoskeleton regulation.

**FIGURE 6:**
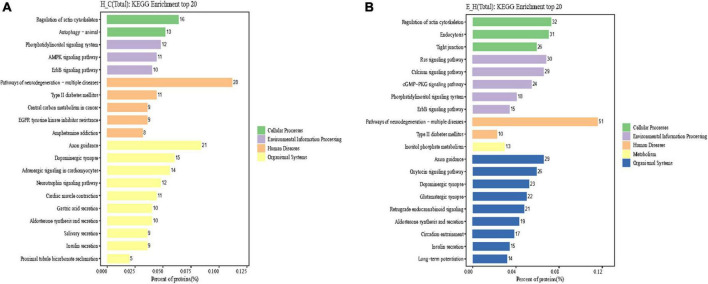
Results of Kyoto Encyclopedia of Genes and Genomes (KEGG) enrichment analysis of differentially phosphorylated proteins in the **(A)** H/C and **(B)** E/H groups. The horizontal axis of the graph presents the ratio (%) of differentially phosphorylated sites corresponding to the expressed proteins annotated to each level of the metabolic pathway to the total number of differentially phosphorylated sites corresponding to the expressed proteins of all differentially phosphorylated sites annotated to the KEGG pathway. The vertical axis represents the name of the level pathway; the number on the right of the column represents the number of differentially phosphorylated sites corresponding to the proteins annotated to that Level pathway, and the columns in different colors represent different levels information.

**FIGURE 7:**
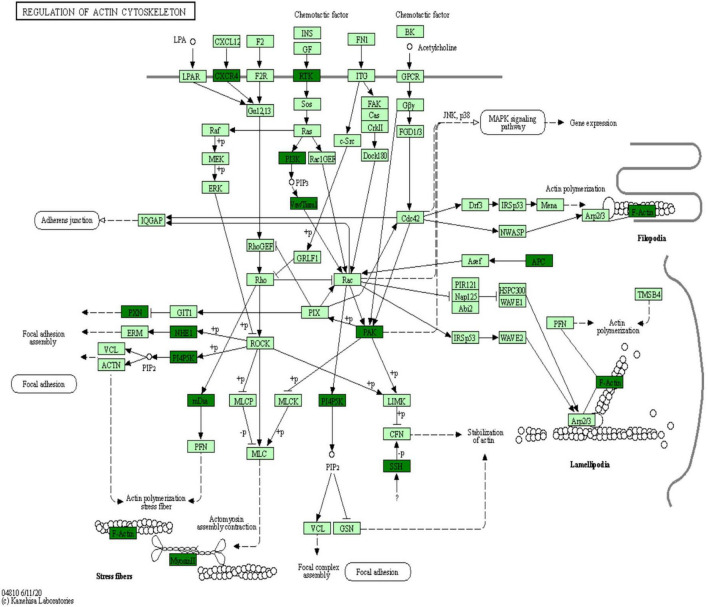
Regulatory signaling pathways of the actin cytoskeleton.

Kyoto Encyclopedia of Genes and Genomes is an information network connecting known molecular interactions such as metabolic pathways, complexes, and biochemical reactions. KEGG pathways mainly include cellular processes, environmental information processing, human diseases, metabolism, organic systems, and drug development (Kanehisa et al., 2017). In the present study, differentially phosphorylated proteins in the H/C and E/H groups were remarkably enriched in the neurodegeneration-multiple disease pathways, followed by actin cytoskeleton regulation and axon guidance signaling pathways, as shown in Figure 6. Notably, the KEGG analysis revealed that MYH10, PAK4, and PIKfyve were involved in actin cytoskeleton regulation (Table 1). Figure 7 shows the regulatory pathways of the actin cytoskeleton.

**TABLE 1:** MYH10, PAK4, and PIKfyve are involved in regulation of actin cytoskeleton pathway.

Group name	Gene name	Regulated type	*P-*value	Positions within proteins	Amino acid	KEGG pathway	Fisher’s exact test *P-*value
H/C	Myh10	Down	1.85253E-05	1,975	S	Regulation of actin cytoskeleton	0.003990707
E/H	Myh10	Up	7.61038E-05	1,975	S	Regulation of actin cytoskeleton	3.23932E-06
H/C	Pak4	Down	8.88122E-06	104	S	Regulation of actin cytoskeleton	0.003990707
E/H	Pak4	Up	5.77836E-07	104	S	Regulation of actin cytoskeleton	3.23932E-06
H/C	PIKfyve	Down	0.003196738	299	S	Regulation of actin cytoskeleton	0.003990707
E/H	PIKfyve	Up	0.000556479	299	S	Regulation of actin cytoskeleton	3.23932E-06
E/H	PIKfyve	Up	1.64413E-06	1,753	S	Regulation of actin cytoskeleton	3.23932E-06

## Discussion

The role of obesity in cognitive dysfunction has become a prominent area of research. Obesity can impair neuronal functions in the brain, leading to behavioral and cognitive deficits evident in animal models. However, the relationship between obesity and cognitive impairment has not been fully elucidated. A recent longitudinal cohort study showed that an SGLT2i was associated with a lower risk of dementia contrasted to the use of an inhibitor of dipeptidyl peptidase 4 (Mui et al., 2021). SGLT2 is are lipid-soluble and can cross the blood-brain barrier, reaching the brain-to-serum area under the curve ratios from 0.3 (dapagliflozin and canagliflozin) to 0.5 (empagliflozin) (Tahara et al., 2016). However, studies on the pathogenesis of obesity-induced cognitive impairment with empagliflozin are lacking. Excitingly, 4-dimensional proteomics is a novel, high-throughput quantitative technology that has emerged to drive the development of precision medicine. In the present study, for the first time, we investigated the effects and action mechanisms of empagliflozin on cognitive functions in the hippocampus of obese mice by performing phosphorylation modification histology in a normal diet, HFD, and empagliflozin-treated mice.

Our results revealed that obese mice fed a high-fat diet had significantly lower levels of SOD and significantly higher levels of MDA compared to normal controls, and that these indices were reversed after engramine treatment. These findings demonstrate that a long-term and regular high-fat diet causes excessive oxidative stress in mice and that administration of exogenous engramine intervention can inhibit and reduce oxidative stress levels. The water maze results showed that high-fat diet-induced obese mice had prolonged escape latency, shortened retention time in the destination quadrant and reduced number of loop penetrations compared to normal controls, and these indices were restored after engramlizine treatment. This suggests that engramlizine has an ameliorative effect on cognitive function, however, the mechanism of its occurrence needs to be further explored. The GO analysis revealed that differentially phosphorylated proteins were mainly involved in actin cytoskeleton regulation. We have described some of them in the following paragraphs.

Encoded by MYH10, non-muscle myosin IIB (NM IIB) belongs to the myosin family and plays a vital role in cell adhesion and migration (Weissenbruch et al., 2021). Studies have shown that myosin II is extensively present in both muscle and non-muscle tissues (e.g., neurons). In muscle tissues, it mainly mediates muscle contraction, whereas, in non-muscle tissues, it primarily maintains the cytoskeleton, also known as NM II (Bi et al., 2015). In addition, NM II plays an important role in the CNS by reversibly binding to actin and hydrolyzing ATP, further conferring a highly dynamic control of neuronal morphology and structure by the cytoskeletal network (Rex et al., 2010). NM II is also involved in the maintenance of long-temporal potentiation, which is closely associated with memory. Ozkan et al. (2015) found that the cell-autonomous deletion of MYH10 leads to specific pathway impairments in dendritic branching development, a structural defect associated with a reduction in the number of functional synapses. In humans, mutations in MYH10 cause a severe CNS phenotype, marked by microcephaly, brain and cerebellar atrophy, and severe intellectual disability (Tuzovic et al., 2013). In the present study, we found for the first time that an HFD affects cytoskeletal remodeling by inhibiting serine phosphorylation at the 1975th site of MYH10, thereby inhibiting neuronal development, and synaptogenesis, leading to decreased cognitive functions in mice. Notably, empagliflozin interfered with this process by increasing serine phosphorylation at the 1975th site of MYH10 to protect cognitive functions.

Protein-activated kinase 4 is a representative member of the class II family of PAKs, which participates in and regulates numerous important cellular processes such as cytoskeletal remodeling, cell transformation, apoptosis, and survival *via* the phosphorylation of downstream substrates (Kumar et al., 2017). Previous studies have shown that the abnormal expression of the PAK4 gene promotes aggressive and invasive metastasis of tumor cells, and targeted therapies against PAK4 can treat several malignancies (Radu et al., 2014). However, recent evidence suggests that PAKs are also critical for neuronal development, axon guidance, and brain-related functions and behaviors (Zhao et al., 2006; Cong et al., 2021). Notably, PAK4 plays a vital role in axonal growth and neuronal development by mediating changes in cell morphology, adhesion, and motility by regulating actin cytoskeleton reorganization in neural progenitor cells (Qu et al., 2003). PAK4 acts as a receptor for cell division control protein 42 (Cdc42) to promote filopodia generation and actin cytoskeleton assembly (Abo et al., 1998), whereas Soosairajah et al. (2005) suggested that PAK4 binds and phosphorylates slingshot, dephosphorylating LIM domain kinase 1 (LIMK1) in the slingshot/LIMKl complex, stimulating actin cytoskeleton reorganization. PAK4-deficient mouse embryos also show damaged axonal growth and neurulation, ultimately leading to growth retardation and premature death (Qu et al., 2003). PAK4, an endogenous neuroprotective kinase, can promote the survival of dopaminergic neurons *via* extracellular signal-regulated kinase- and AKT-regulated signaling pathways. A decrease in PAK4 levels and activity in the human brain during aging can cause a pre-Parkinsonian state (Kuijl et al., 2007). In the present study, for the first time, we observed that an HFD-induced decrease in PAK4 phosphorylation at Ser104 affected the actin cytoskeleton assembly, leading to a decrease in cognitive functions in the mice, which was consistent with the above-mentioned studies. However, PAK4 phosphorylation at the Ser104 site was promoted after empagliflozin treatment to restore this process for protecting cognitive functions.

Phosphatidylinositol 3-phosphate 5-kinase is the only lipid kinase in mammals that catalyzes the production of phosphatidylinositol-3,5-bisphosphate [PI(3,5)P2] from phosphatidylinositol-3-phosphate and plays a role in exosome release, endocytic pathway, cell migration, and autophagy (Lees et al., 2020). In contrast, the inactivation of PIKfyve or its regulators Fig4/Sac3 and Vac14/ArPIKfyve leads to various physiological problems, including embryonic death, neurodegeneration, and immune dysfunction. In animal studies, mutations in Fig4 or Vac14 resulted in reduced PI(3,5)P2 levels and cell vacuolization in the CNS with marked spongiform degeneration in mice (Chow et al., 2007; Zhang et al., 2007). Lysosomes have been reported to mediate activity-dependent Ca^2^ + signaling in the dendrites and improve memory by maintaining neuronal structural plasticity (Tsuruta and Dolmetsch, 2015). PIKfyve is a key regulator of endolysosomal system homeostasis, and PIKfyve inhibition leads to impaired cellular degradability, ion dysregulation, diminished autophagic flux, and enlarged lysosome volume (Choy et al., 2018). Chronic intermittent hypoxia can downregulate the adenosine monophosphate-activated protein kinase-PIKfyve-PtdIns (3,5)P2 pathway, ultimately impairing lysosomal functions (Fernandez-Mosquera et al., 2019). Martin et al. (2013) showed that the use of YM-201636 inhibited PIKfyve kinase activity, dysregulating autophagy and consequently causing hippocampal neuronal cell death. In the present study, we found that the HFD-induced reduction of PIKfyve phosphorylation at Ser299 may impair cellular degradation and diminish autophagic flux, resulting in neuronal damage. Notably, empagliflozin improved memory by regulating the increased phosphorylation of Ser299 and 1,753 at PIKfyve, promoting the fusion of autophagosomes and lysosomes, and attenuating neural damage and neuronal death.

The KEGG analysis of the phosphorylated proteins was also performed in this study. The results indicated that the phosphorylated proteins were enriched in neurodegeneration-multiple disease pathways, actin cytoskeleton regulation, and axon guidance signaling pathways (Figure 6). Importantly, MYH10, PAK4, and PIKfyve were involved in actin cytoskeleton regulation according to the KEGG analysis (Table 1). We showed that synaptic plasticity, which mainly includes functional plasticity and morphological plasticity, is an important mechanism of learning memory. On the other hand, actin cytoskeleton regulation is the structural basis of synaptic morphological plasticity (mainly involving changes in synaptic morphology, synaptic density, and dendritic spine density) and the basis of the hippocampal neuron structure and function. In contrast, actin polymerization, depolymerization, and remodeling are regulated by actin-binding proteins and upstream signaling molecules. MYH10, a cytoskeleton regulatory protein, is involved in the dynamic regulation of the cytoskeleton *via* the reversible binding to actin (Martin et al., 2013). PAK4, a key signaling node in the small molecule GTPase CDC42/ribosome-associated complex-PAK signaling pathway, regulates slingshot by phosphorylating cytoskeletal rearrangement (Soosairajah et al., 2005). PIKfyve, a protein kinase, is important in controlling the nervous system and brain environment and can be involved in cytoskeletal rearrangement by regulating microfilament polymerization/depolymerization (Wu et al., 2017).

Our results revealed that a high-fat diet increased the levels of TC, TG, LDL-C, and HDL-C in mice, and that after the intervention of empagliflozin, the levels of TC, TG, and LDL-C decreased along with a significant decrease in HDL-C. Previous studies have shown that cardiovascular risk decreases with increasing levels of high-density lipoprotein cholesterol (HDL-C) (Liu et al., 2022). However, recent studies suggest that the relationship between HDL-C and cardiovascular events may be a U-shaped curve relationship, i.e., too high and too low is bad (Hamer et al., 2018). Zanoni et al. (2016) found that in people with deletion of the SCARB1 gene (Scavenger receptor BI; its product is the main receptor for HDL-C), although HDL-C concentrations were significantly higher than normal, they had a higher risk of cardiovascular disease than normals. It has also been shown that dysfunctional HDL contains high levels of pro-oxidant molecules that prevent HDL transport to eliminate metabolic waste and increase the risk of coronary heart disease (Franczyk et al., 2021). Based on the results of the present study, we hypothesized that a high-fat diet may lead to HDL dysfunction and promote the development of inflammatory responses, and that empagliflozin may reduce its levels and improve oxidative stress, thereby improving glucolipid metabolism. Compared to normal controls, obese mice fed a high-fat diet had significantly lower levels of SOD and significantly higher levels of MDA, and the above indices were reversed after empagliflozin treatment. These findings demonstrate that a long-term and regular high-fat diet causes excessive oxidative stress in mice, and the administration of exogenous empagliflozin intervention can inhibit and reduce oxidative stress levels. The water maze results showed that the escape latency was prolonged, the retention time in the destination quadrant was shortened and the number of loop penetrations was reduced in obese mice induced by high-fat diet compared with normal controls, while the above indices were restored after empagliflozin treatment. This suggests that empagliflozin has an improving effect on cognitive function, however, its mechanism of action still needs to be further explored. SGLT2i is known to be a novel hypoglycemic agent with pleiotropic properties such as hypoglycemia and improvement of cardiovascular and renal vascular outcomes. However, there is growing evidence that SGLT2 inhibitors have neuroprotective potential. A case-control study including 176 250 patients with type 2 diabetes showed that SGLT2 inhibitors were associated with a lower OR for dementia (Wium-Andersen et al., 2019). A randomized, double-blind, placebo-controlled study by Kullmann et al. (2022) found that the SGLT2 inhibitor empagliflozin has the potential to be used to treat insulin resistance in the brain, while having a positive effect on reducing body fat and improving systemic metabolism effects. Therefore, we hypothesize that the benefits of SGLT2 inhibitors could be extended to these patients with cognitive impairment, but more future clinical studies are needed to further confirm this. The GO analysis revealed that differentially phosphorylated proteins were mainly involved in actin cytoskeleton regulation. We have described some of them in the following paragraphs.

## Conclusion

In the present study, for the first time, we showed that the phosphorylation levels of MYH10, PAK4, and PIKfyve, which are involved in actin cytoskeleton regulation, decreased in mice fed with an HFD and increased after the empagliflozin intervention. Therefore, we hypothesized that MYH10, PAK4, and PIKfyve might be the targets of obesity-induced cognitive impairment, providing new ideas, and directions to understand the pathogenesis of obesity-induced cognitive impairment. Though the preliminary results of this study support this hypothesis, this study has the following limitations: (1) Hippocampal tissue samples are difficult to obtain, and the volume of individual samples is small for a single-sample analysis; (2) The study was performed using only hippocampal tissues, and the sample types were single. (3) Due to the limitations of the sample size, time, and experimental conditions, we were unable to verify the functions of the differentially expressed and phosphorylated modified proteins in this study. In our next study, we will perform more experiments to confirm the target relationships between genes and pathways and their interacting functions.

## Data availability statement

The datasets presented in this study can be found in online repositories. The names of the repository/repositories and accession number(s) can be found in the article/Supplementary material.

## Ethics statement

This animal study was reviewed and approved by the Animal Ethics Committee of Hebei General Hospital (No. 202173).

## Author contributions

SC designed the study. XC, LM, JZ, and XP performed the experiments and analyzed the data. All authors wrote the manuscript and approved the final version of the manuscript.

## References

[B1] Abo A. Qu J. Cammarano M. S. Dan C. Fritsch A. Baud V. (1998). PAK4, a novel effector for Cdc42Hs, is implicated in the reorganization of the actin cytoskeleton and in the formation of filopodia. *EMBO J.* 17 6527–6540. 10.1093/emboj/17.22.6527 9822598 PMC1171000

[B2] Bi A. L. Wang Y. Zhang S. Li B. Q. Sun Z. P. Bi H. S. (2015). Myosin II regulates actin rearrangement-related structural synaptic plasticity during conditioned taste aversion memory extinction. *Brain Struct. Funct.* 220 813–825. 10.1007/s00429-013-0685-5 24337340

[B3] Chow C. Y. Zhang Y. Dowling J. J. Jin N. Adamska M. Shiga K. (2007). Mutation of FIG4 causes neurodegeneration in the pale tremor mouse and patients with CMT4J. *Nature* 448 68–72. 10.1038/nature05876 17572665 PMC2271033

[B4] Choy C. H. Saffi G. Gray M. A. Wallace C. Dayam R. M. Ou Z. A. (2018). Lysosome enlargement during inhibition of the lipid kinase PIKfyve proceeds through lysosome coalescence. *J. Cell Sci.* 131:jcs213587. 10.1242/jcs.213587 29661845 PMC6031331

[B5] Cong C. Liang W. Zhang C. Wang Y. Yang Y. Wang X. (2021). PAK4 suppresses motor neuron degeneration in hSOD1(G93A) -linked amyotrophic lateral sclerosis cell and rat models. *Cell Prolif.* 54:e13003. 10.1111/cpr.13003 33615605 PMC8016643

[B6] Fernandez-Mosquera L. Yambire K. F. Couto R. Pereyra L. Pabis K. Ponsford A. H. (2019). Mitochondrial respiratory chain deficiency inhibits lysosomal hydrolysis. *Autophagy* 15 1572–1591. 10.1080/15548627.2019.1586256 30917721 PMC6693470

[B7] Franczyk B. Rysz J. Lawinski J. Rysz-Gorzynska M. Gluba-Brzozka A. (2021). Is a high HDL-cholesterol level always beneficial? *Biomedicines* 9:1083. 10.3390/biomedicines9091083 34572269 PMC8466913

[B8] Hamer M. O’Donovan G. Stamatakis E. (2018). High-density lipoprotein cholesterol and mortality: Too much of a good thing? *Arterioscler. Thromb. Vasc. Biol.* 38 669–672. 10.1161/ATVBAHA.117.310587 29326314

[B9] Im D. S. Joselin A. Svoboda D. Takano T. Rousseaux M. Callaghan S. (2022). Cdk5-mediated JIP1 phosphorylation regulates axonal outgrowth through Notch1 inhibition. *BMC Biol.* 20:115. 10.1186/s12915-022-01312-4 35581583 PMC9115922

[B10] Kamacioglu A. Tuncbag N. Ozlu N. (2021). Structural analysis of mammalian protein phosphorylation at a proteome level. *Structure* 29 1219–1229. 10.1016/j.str.2021.06.008 34192515

[B11] Kanehisa M. Furumichi M. Tanabe M. Sato Y. Morishima K. (2017). KEGG: New perspectives on genomes, pathways, diseases and drugs. *Nucleic Acids Res.* 45 D353–D361. 10.1093/nar/gkw1092 27899662 PMC5210567

[B12] Kuijl C. Savage N. D. Marsman M. Tuin A. W. Janssen L. Egan D. A. (2007). Intracellular bacterial growth is controlled by a kinase network around PKB/AKT1. *Nature* 450 725–730. 10.1038/nature06345 18046412

[B13] Kullmann S. Hummel J. Wagner R. Dannecker C. Vosseler A. Fritsche L. (2022). Empagliflozin improves insulin sensitivity of the hypothalamus in humans with prediabetes: A randomized, double-blind, placebo-controlled, phase 2 trial. *Diabetes Care* 45 398–406. 10.2337/dc21-1136 34716213 PMC8914418

[B14] Kumar R. Sanawar R. Li X. Li F. (2017). Structure, biochemistry, and biology of PAK kinases. *Gene* 605 20–31. 10.1016/j.gene.2016.12.014 28007610 PMC5250584

[B15] Lee J. K. Kim N. J. (2017). Recent advances in the inhibition of p38 MAPK as a potential strategy for the treatment of Alzheimer’s disease. *Molecules* 22:1287. 10.3390/molecules22081287 28767069 PMC6152076

[B16] Lees J. A. Li P. Kumar N. Weisman L. S. Reinisch K. M. (2020). Insights into lysosomal PI(3,5)P2 homeostasis from a structural-biochemical analysis of the PIKfyve lipid kinase complex. *Mol. Cell* 80 736–743. 10.1016/j.molcel.2020.10.003 33098764 PMC7962480

[B17] Lin B. Koibuchi N. Hasegawa Y. Sueta D. Toyama K. Uekawa K. (2014). Glycemic control with empagliflozin, a novel selective SGLT2 inhibitor, ameliorates cardiovascular injury and cognitive dysfunction in obese and type 2 diabetic mice. *Cardiovasc. Diabetol.* 13:148. 10.1186/s12933-014-0148-1 PMC4219031 25344694

[B18] Lin H. Caroll K. S. (2018). Introduction: Posttranslational protein modification. *Chem. Rev.* 118 887–888. 10.1021/acs.chemrev.7b00756 29439579

[B19] Liu C. Dhindsa D. Almuwaqqat Z. Sun Y. V. Quyyumi A. A. (2022). Very high high-density lipoprotein cholesterol levels and cardiovascular mortality. *Am. J. Cardiol.* 167 43–53. 10.1016/j.amjcard.2021.11.041 35039162

[B20] Martin S. Harper C. B. May L. M. Coulson E. J. Meunier F. A. Osborne S. L. (2013). Inhibition of PIKfyve by YM-201636 dysregulates autophagy and leads to apoptosis-independent neuronal cell death. *PLoS One* 8:e60152. 10.1371/journal.pone.0060152 23544129 PMC3609765

[B21] Miller A. A. Spencer S. J. (2014). Obesity and neuroinflammation: A pathway to cognitive impairment. *Brain Behav. Immun.* 42 10–21. 10.1016/j.bbi.2014.04.001 24727365

[B22] Mui J. V. Zhou J. Lee S. Leung K. Lee T. Chou O. (2021). Sodium-glucose cotransporter 2 (SGLT2) inhibitors vs. dipeptidyl peptidase-4 (DPP4) inhibitors for new-onset dementia: A propensity score-matched population-based study with competing risk analysis. *Front. Cardiovasc. Med.* 8:747620. 10.3389/fcvm.2021.747620 34746262 PMC8566991

[B23] Ozkan E. D. Aceti M. Creson T. K. Rojas C. S. Hubbs C. R. McGuire M. N. (2015). Input-specific regulation of hippocampal circuit maturation by non-muscle myosin IIB. *J. Neurochem.* 134 429–444. 10.1111/jnc.13146 25931194 PMC4496335

[B24] Ponasenko A. Sinitsky M. Minina V. Vesnina A. Khutornaya M. Prosekov A. (2022). Immune response and lipid metabolism gene polymorphisms are associated with the risk of obesity in middle-aged and elderly patients. *J. Pers. Med.* 12:238. 10.3390/jpm12020238 35207726 PMC8879873

[B25] Putz S. M. Kram J. Rauh E. Kaiser S. Toews R. Lueningschroer-Wang Y. (2021). Loss of p21-activated kinase Mbt/PAK4 causes Parkinson-like phenotypes in *Drosophila*. *Dis. Models Mech.* 14:dmm047811. 10.1242/dmm.047811 34125184 PMC8246267

[B26] Qu J. Li X. Novitch B. G. Zheng Y. Kohn M. Xie J. M. (2003). PAK4 kinase is essential for embryonic viability and for proper neuronal development. *Mol. Cell. Biol.* 23 7122–7133. 10.1128/MCB.23.20.7122-7133.2003 14517283 PMC230313

[B27] Radu M. Semenova G. Kosoff R. Chernoff J. (2014). PAK signalling during the development and progression of cancer. *Nat. Rev. Cancer* 14 13–25. 10.1038/nrc3645 24505617 PMC4115244

[B28] Rex C. S. Gavin C. F. Rubio M. D. Kramar E. A. Chen L. Y. Jia Y. (2010). Myosin IIb regulates actin dynamics during synaptic plasticity and memory formation. *Neuron* 67 603–617. 10.1016/j.neuron.2010.07.016 20797537 PMC2929390

[B29] Rivero-Rios P. Weisman L. S. (2022). Roles of PIKfyve in multiple cellular pathways. *Curr. Opin. Cell Biol.* 76:102086. 10.1016/j.ceb.2022.102086 35584589 PMC9108489

[B30] Sa-Nguanmoo P. Tanajak P. Kerdphoo S. Jaiwongkam T. Pratchayasakul W. Chattipakorn N. (2017). SGLT2-inhibitor and DPP-4 inhibitor improve brain function via attenuating mitochondrial dysfunction, insulin resistance, inflammation, and apoptosis in HFD-induced obese rats. *Toxicol. Appl. Pharmacol.* 333 43–50. 10.1016/j.taap.2017.08.005 28807765

[B31] Solas M. Milagro F. I. Ramirez M. J. Martinez J. A. (2017). Inflammation and gut-brain axis link obesity to cognitive dysfunction: Plausible pharmacological interventions. *Curr. Opin. Pharmacol.* 37 87–92. 10.1016/j.coph.2017.10.005 29107872

[B32] Soosairajah J. Maiti S. Wiggan O. Sarmiere P. Moussi N. Sarcevic B. (2005). Interplay between components of a novel LIM kinase-slingshot phosphatase complex regulates cofilin. *EMBO J.* 24 473–486. 10.1038/sj.emboj.7600543 15660133 PMC548651

[B33] Tahara A. Takasu T. Yokono M. Imamura M. Kurosaki E. (2016). Characterization and comparison of sodium-glucose cotransporter 2 inhibitors in pharmacokinetics, pharmacodynamics, and pharmacologic effects. *J. Pharmacol. Sci.* 130 159–169. 10.1016/j.jphs.2016.02.003 26970780

[B34] Tsuruta F. Dolmetsch R. E. (2015). PIKfyve mediates the motility of late endosomes and lysosomes in neuronal dendrites. *Neurosci. Lett.* 605 18–23. 10.1016/j.neulet.2015.07.021 26232680

[B35] Tuzovic L. Yu L. Zeng W. Li X. Lu H. Lu H. M. (2013). A human de novo mutation in MYH10 phenocopies the loss of function mutation in mice. *Rare Dis.* 1:e26144. 10.4161/rdis.26144 25003005 PMC3927488

[B36] Weissenbruch K. Grewe J. Hippler M. Fladung M. Tremmel M. Stricker K. (2021). Distinct roles of nonmuscle myosin II isoforms for establishing tension and elasticity during cell morphodynamics. *Elife* 10:e71888. 10.7554/eLife.71888 34374341 PMC8391736

[B37] Wium-Andersen I. K. Osler M. Jorgensen M. B. Rungby J. Wium-Andersen M. K. (2019). Antidiabetic medication and risk of dementia in patients with type 2 diabetes: A nested case-control study. *Eur. J. Endocrinol.* 181 499–507. 10.1530/EJE-19-0259 31437816

[B38] Wu Y. Zhuang J. Zhao D. Zhang F. Ma J. Xu C. (2017). Cyclic stretch-induced the cytoskeleton rearrangement and gene expression of cytoskeletal regulators in human periodontal ligament cells. *Acta Odontol. Scand.* 75 507–516. 10.1080/00016357.2017.1347823 28681629

[B39] Xiao H. Ma X. J. Cheng O. M. Qiu H. M. Jiang Q. S. (2019). [Gastrodin improves hippocampal neurogenesis by NO-cGMP-PKG signaling pathway in cerebral ischemic mice]. *Zhongguo Zhong Yao Za Zhi* 44 5451–5456. 10.19540/j.cnki.cjcmm.20190819.401 32237394

[B40] Yu A. S. Hirayama B. A. Timbol G. Liu J. Diez-Sampedro A. Kepe V. (2013). Regional distribution of SGLT activity in rat brain in vivo. *Am. J. Physiol. Cell Physiol.* 304 C240–C247. 10.1152/ajpcell.00317.2012 23151803 PMC3566441

[B41] Yu J. T. Xu W. Tan C. C. Andrieu S. Suckling J. Evangelou E. (2020). Evidence-based prevention of Alzheimer’s disease: Systematic review and meta-analysis of 243 observational prospective studies and 153 randomised controlled trials. *J. Neurol. Neurosurg. Psychiatry* 91 1201–1209. 10.1136/jnnp-2019-321913 32690803 PMC7569385

[B42] Zanoni P. Khetarpal S. A. Larach D. B. Hancock-Cerutti W. F. Millar J. S. Cuchel M. (2016). Rare variant in scavenger receptor BI raises HDL cholesterol and increases risk of coronary heart disease. *Science* 351 1166–1171. 10.1126/science.aad3517 26965621 PMC4889017

[B43] Zhang Y. Zolov S. N. Chow C. Y. Slutsky S. G. Richardson S. C. Piper R. C. (2007). Loss of Vac14, a regulator of the signaling lipid phosphatidylinositol 3,5-bisphosphate, results in neurodegeneration in mice. *Proc. Natl. Acad. Sci. U.S.A.* 104 17518–17523. 10.1073/pnas.0702275104 17956977 PMC2077288

[B44] Zhao L. Ma Q. L. Calon F. Harris-White M. E. Yang F. Lim G. P. (2006). Role of p21-activated kinase pathway defects in the cognitive deficits of Alzheimer disease. *Nat. Neurosci.* 9 234–242. 10.1038/nn1630 16415866

[B45] Zinman B. Wanner C. Lachin J. M. Fitchett D. Bluhmki E. Hantel S. (2015). Empagliflozin, cardiovascular outcomes, and mortality in type 2 diabetes. *N. Engl. J. Med.* 373 2117–2128. 10.1056/NEJMoa1504720 26378978

